# The mediating role of step counts in the relationship between diverse neighbourhood destinations and older adults’ physical function

**DOI:** 10.1038/s41598-024-80699-2

**Published:** 2025-04-11

**Authors:** Chien-Yu Lin, Hsin-Yen Yen, Ming-Chun Hsueh, Jong-Hwan Park, Yung Liao

**Affiliations:** 1 Department of Public Health, College of Medicine, National Cheng Kung University, Tainan, Taiwan; 2 Faculty of Sport Sciences, Waseda University, Tokorozawa, Japan; 3 Centre for Urban Transitions, Swinburne University of Technology, Melbourne, Australia; 4 School of Gerontology Health Management, College of Nursing, Taipei Medical University, Taipei, Taiwan; 5 Graduate Institute of Sport Pedagogy, University of Taipei, Taipei, Taiwan; 6 Master’s Program of Transition and Leisure Education for Individuals with Disabilities, University of Taipei, Taipei, Taiwan; 7 Convergence Medical Institute of Technology, Pusan National University Hospital, 179, Gudeok-ro, Seo-gu, Busan, 49241 South Korea; 8 Department of Convergence Medicine, Pusan National University School of Medicine, Yangsan, South Korea; 9 Department of Clinical Bio-Convergence, Graduate School of Convergence in Biomedical Science, Pusan National University School of Medicine, Yangsan, South Korea; 10 Graduate Institute of Sport, Leisure and Hospitality Management, College of Sports and Recreation, National Taiwan Normal University, 6F, No 129-1, Section 1, Heping East Road, Da’an District, Taipei City 106, Taiwan

**Keywords:** Elderly, Functional test, Urban design, Walkability, Destination, Disease prevention, Geriatrics, Public health

## Abstract

Research has shown that access to activity-supportive neighbourhood destinations is associated with physical activity levels and physical functioning in older adults. However, the relative importance of different destinations and the mechanisms underlying these relationships remain unclear. We investigated (i) the relationships between the availability of diverse neighbourhood destinations and physical functioning in Taiwanese older adults and (ii) whether step counts mediated these relationships. Data were analysed from 206 participants aged 65 years or above, recruited from geriatrics outpatient clinics or check-up services at a medical centre in Taipei, Taiwan. Geographic information system technology was used to assess the availability of utilitarian destinations, public transport stations, parks, schools, and sports facilities based on geocoded addresses. Physical functioning was evaluated through on-site examinations. Logistic and linear regression models were used to investigate the relationships and joint-significance tests were used to examine mediation effects. The participants (52.4% women) were generally healthy, with no severe comorbidities, good cognitive function, and a low risk of depression. Adjusted models showed that greater park availability was associated with better physical functioning, including upper and lower limb strength and mobility. The relationship appeared to be partially mediated by increased step counts. Some evidence suggested that walking steps also mediated the relationship between the availability of public transport stations and physical functioning. However, no direct relationhips were found between public transport station availability and physical functioning, suggesting potential offseting factors. These findings suggest strategic allocation of neighbourhood resources, such as parks, to promote walking behaviour and enhance physical functioning in older adults. Future research should explore park design, usage patterns, and other potential mediators related to public transport stations to refine strategies for maintaining and improving physical functioning in ageing populations.

**Supplementary Information:**

The online version contains supplementary material available at 10.1038/s41598-024-80699-2.

## Introduction

The world’s population is ageing, with the proportion of older adults steadily increasing. In 2019, individuals aged 65 years or above accounted for 9% of the global population, a figure projected to rise to 16% in 2050^1^. This demographic shift is expected to lead to greater difficulties in physical functioning among older adults^2,3^, reducing their quality of life^4^ and incurring substantial economic and social costs^5^. Physical functioning, defined as the ability to perform basic and instrumental daily activities independently, encompasses several interrelated subdomains such as muscle strength, mobility, and endurance^6^.

Extant evidence indicates that the availability or accessibility of neighbourhood destinations is associated with better physical functioning, including balance^7^ and mobility^8,9^. A systematic review has suggested that physical activity may mediate the relationship between the built environment and physical functioning^10^. It is postulated that daily step counts partially explain the relationship between the availability of neighbourhood destinations and physical functioning. Previous studies have found that higher walking steps are associated with greater destination density or easier access to neighbourhood amenities^11^ and contribute to improved physical functioning^12^.

One study examined the potential mediating role of physical activity in the relationship between neighbourhood walkability and physical health, including physical functioning, but found no mediation^13^. However, its generalisability is limited, as it targeted older adults hospitalised for acute myocardial infarction rather than the general ageing population. Additionally, physical health in the previous study included not only physical functioning but also physical activities, pain, and general health. The concept of neighbourhood walkability in the previous research included various types of destinations. A review has suggested that different neighbourhood destinations are associated with adults’ walking behaviours for different purposes^14^, yet it remains unclear whether the pathway from destination availability to physical functioning through walking behaviours varies by destination type.

This study investigated (1) the relationships between the availability of different neighbourhood destinations and indicators of physical functioning and (2) whether these relationships are mediated by walking steps, using a sample of Taiwanese adultsaged 65 years or above.

## Methods

### Participants

This study was cross-sectional design. Older adults aged 65 years or above were recruited from the geriatrics and gerontology outpatient clinics or community check-up services at National Taiwan University Hospital, a medical centre in Taipei City, Taiwan, between September 2020 and December 2021. Invited participants were primarily screened by physicians or research assistants to ensure they could understand the study procedures and instructions. A total of 301 participants completed structured questionnaires, providing data on demographic characteristics and current residential addresses. All participants completed on-site physical functioning assessments and wore accelerometers for seven consecutive days to record step counts. After excluding participants with incomplete residential addresses (*n* = 13), missing physical functioning data (*n* = 40), invalid accelerometer data (*n* = 38), or missing demographic/health condition data (*n* = 4), 206 eligible older adults were included in the complete-case analyses. Missing or invalid data were assumed to be missing at random and imputed in a sensitivity analysis. Each participant signed an informed consent form before starting the survey and received a US$7 gift voucher for their participation. This study was approved by the Research Ethics Committee of the National Taiwan University Hospital (202008046RINC) and performed in accordance with the Declaration of Helsinki.

### Exposures: availability of neighbourhood destinations

Data on various destinations, including utilitarian destinations, public transport stations, parks, sports facilities, and schools, were extracted from the National Land Surveying and Mapping Center, Ministry of the Interior (https://maps.nlsc.gov.tw/). These destinations were chosen based on their relevance to older adults’ physical activities. Utilitarian destinations included supermarkets, retail stores, department stores, post offices, and banks, where older adults may travel for daily errands. Public transport stations included bus, train, metro, tram, and high-speed rail stations. For leisure activities, parks, schools, and sports facilities were identified. Open spaces such as parks and school playgrounds are commonly used for physical activity among older adults in Taiwan^15.^ Since 2012, the Taipei City Government has encouraged schools to open their campuses to promote active lifestyles, and thus schools became key destinations for walking and physical activity^16^. Parks and school playgrounds were distinguished based on their facilities and availability times, while sports facilities such as gyms, stadiums, and swimming pools were designated for higher-intensity activities. Neighbourhoods were defined as a 400-metre circular buffer around each participant’s geocoded residential address, using ESRI ArcGIS Desktop 10 software. This buffer zone was selected according to a previous review of residential built environments and health^17^. To preserve statistical power, the number of destinations within each neighbourhood was summed and log-transformed due to its skewed distribution, rather than categorised into binary variables (i.e., low vs. high availability)^10,18^.

### Outcome: physical functioning

Physical functioning was assessed through three on-site examinations measuring upper limb strength, lower limb strength, and mobility. Upper limb strength was measured using handgrip strength. Participants were instructed to use one hand to squeeze the hydraulic hand-held dynamometer (Jamar Plus + Digital Hand Dynamometer, Lafayette Instrument Company, USA) with maximum effort while standing and maintain the position for three seconds. Two attempts were conducted with a one-minute rest between attempts and the higher value in kilogram was recorded. Lower limb strength was assessed using the five-time sit-to-stand test, in which participants were timed as they stood up and sat down five times. The shorter completion time from two attempts was recorded. Mobility was measured by usual gait speed. Participants walked a six-metre straight path twice, and the shorter time was recorded. Professionals walked alongside participants and recorded the time in seconds using standardised procedures.

Based on the Asian Working Group for Sarcopenia (AWGS) 2019 report^19^, we divided handgrip strength (<28 kg for men or <18 kg for women as poor upper limb strength), five-time sit-to-stand test (≥12 s as poor lower limb strength), and gait speed (<1.0 m/s as poor mobility) into two categories. Muscle mass was not included in this study due to lack of variation among participants (>95% of the participants showed ideal performance).

### Potential mediating factor: step counts

Step counts were measured using triaxial accelerometers (ActiGraph, Pensacola, FL, USA), which participants wore for seven consecutive days (i.e., five weekdays and two weekend days). The ActiGraph accelerometer is widely used and validated for physical activity monitoring^20^. Participants were instructed to wear the device on the right side of their waist throughout the day, except during water-based activities. Valid data required at least three days of wear, including one weekend day, with a minimum of 10 hours per day. Non-wear time was defined as 60 continuous minutes of zero counts per minute, allowing up to 2 minutes of slight movement (<100 counts per minute)^22^. Non-wear time was cross-verified with a daily log sheet, where participants recorded the times they put on and removed the device^21^. Only participants with at least three valid days of data, including one weekend day, were included in the analysis. Accelerometer data, recorded in 60-second epochs, were processed using ActiLife software 6.0 (Pensacola, FL, USA). Step counts per minute were calculated by dividing total step counts by wear time.

### Covariates

Demographic characteristics and health indicators were collected via questionnaires. Demographic characteristics included gender, age group (60–74, 75–84, and 85 years or above), and education level (high school or lower vs university or above). Health conditions included chronic comorbidities, cognitive function, and depression. Chronic diseases such as type 2 diabetes, cerebrovascular diseases, cardiovascular diseases, chronic respiratory diseases, liver diseases, renal diseases, neurodegenerative disorders, neoplasia, haematological diseases, and immune diseases, were self-reported based on the Charlson Comorbidity Index^23^. Severe comorbidity was defined as having ≥4 chronic diseases, as individuals with this level of multimorbidity are approximately 100 times more likely to require ambulatory care hospitalisation than those without chronic conditions^24^. Cognitive function was assessed using the Mini-Mental State Examination, a widely used screening tool for older adults with good test-retest reliability (0.80–0.95) and acceptable sensitivity and specificity^25^. Cognitive function was classified as “good” or “poor”, with cutoffs adjusted for educational level. Those who identified as having “good” cognitive function had a score of >16 (without formal education), >20 (elementary education), or >23 (high school or above)^26^. Depression was measured using the Geriatric Depression Scale-15, which showed an acceptable pooled sensitivity (0.80) and specificity (0.79)^27^. Depression levels were categorised as “healthy (≤5)”, “mildly depressed (6–9)”, and “depressed (≥10)”^28^. Seasonal variations in walking behaviours were also considered in the models.

### Data analyses

Descriptive analyses were performed to summarise participant characteristics, presented as counts and percentages. Chi-squared tests were used to compare characteristics between different study sites. Since multicollinearity was detected among destination availability variables (variance inflation factor >5)^29^, each destination type was included in separate models.

We first examined the total effect, assessing the relationships between the availability of a range of neighbourhood destinations and indicators of physical functioning. Afterwards, we investigated the relationship between destination availability and step counts (α) and the relationship between step counts and physical functioning (β). Logistic regression was used to estimate total effects and β coefficients, while linear regression was applied to estimate α coefficients. Each type of destination was included in separate models, with adjusted odds ratios (ORs) or unstandardised coefficients (B) and their 95% confidence intervals (CIs) estimated. Reference groups consisted of participants with below-median destination availability and those classified as having better physical functioning based on AWGS criteria. The ORs below one were interpreted as lower odds of physical dysfunction.

Joint-significance tests were used to examine mediation effects, which were identified when both coefficients α and β were statistically significant^30^. This approach is widely used in health research^31–33^. A bootstrapping mediation analysis was not performed due to the small sample size, which may yield unstable estimates^34^. We conducted a sensitivity analysis using multiple imputation techniques to account for missing data to assess the robustness of findings. The multiple imputation method generated multiple several imputed datasets and estimates were pooled using Rubin’s rules^35^, which account for both within- and between-imputation variance. Note that the term “effect” is used throughout the study in a statistical sense, and does not imply causality. Sensitivity analyses were conducted using 500- and 800-m circular buffers around each participant’s geocoded residential address to examine the robustness of findings. All analyses were conducted using SPSS version 22.0 (IBM Corp, Armonk, NY). The level of significance was set at 0.05.

## Results

Tables 1 and 2 show the characteristics of participants with complete data. Most participants were recruited from outpatient clinics, were women, and were relatively healthy, without severe chronic comorbidities. They also had good cognitive function and a low risk of depression (Table 1). Participants from outpatient clinics were older, less educated, and had lower cognitive, mental, and physical functioning than those recruited from community check-ups. The distribution of neighbourhood destinations and step counts was shown in Table 2.

**Table 1:** Characteristics of study participants (*n* = 206).

	Total		Study site				
			Community for check-ups (*n* = 77)		Outpatient clinics (*n* = 129)		*p*
	N	(%)	N	(%)	N	(%)	
Sex							0.33
Women	108	(52.4)	37	(48.1)	71	(55.0)	
Men	98	(47.6)	40	(51.9)	58	(45.0)	
Age group							< 0.001
60–74 years	100	(48.5)	47	(61.0)	53	(41.1)	
75–84 years	82	(39.8)	30	(39.0)	52	(40.3)	
85+ years	24	(11.7)	0	(0.0)	24	(18.6)	
Education							< 0.001
High school or lower	103	(50.0)	19	(24.7)	84	(65.1)	
University or above	103	(50.0)	58	(75.3)	45	(34.9)	
Chronic comorbidity							0.24
Low (< 4 diseases)	183	(88.8)	71	(92.2)	112	(86.8)	
High (≥ 4 diseases)	23	(11.2)	6	(7.8)	17	(13.2)	
Cognitive function							< 0.001
Good	183	(88.8)	77	(100.0)	106	(82.2)	
Poor	23	(11.2)	0	(0.0)	23	(17.8)	
Depression							< 0.001
Healthy	171	(83.0)	76	(98.7)	95	(73.6)	
Mild depressed	23	(11.2)	1	(1.3)	22	(17.1)	
Depressed	12	(5.8)	0	(0.0)	12	(9.3)	
Mobility							< 0.001
Good	122	(59.2)	72	(93.5)	50	(38.8)	
Poor	84	(40.8)	5	(6.5)	79	(61.2)	
Lower limb strength							< 0.001
Good	111	(53.9)	72	(93.5)	39	(30.2)	
Poor	95	(46.1)	5	(6.5)	90	(69.8)	
Upper limb strength							< 0.001
Good	127	(61.7)	70	(90.9)	57	(44.2)	
Poor	79	(38.3)	7	(9.1)	72	(55.8)	

**Table 2:** Distribution of neighbourhood destinations within 400-m buffer of participants’ residence and step counts.

	Mean	SD	Min	Median	Max
Neighbourhood destination					
Utilitarian destination	23.0	15.4	0.0	21.0	100.0
Public transport station	2.3	3.1	0.0	0.0	13.0
Park	3.3	2.4	0.0	3.0	10.0
Sports facility	0.4	0.8	0.0	0.0	6.0
School	2.6	2.3	0.0	2.0	10.0
Step (counts/minute)	6.0	3.9	0.4	5.8	31.9

SD: standard deviation.

Note: A binary variable of each neighbourhood destination (low vs. high availability) was categorised based on their median.

A higher level of park availability in residential neighbourhoods was associated with a lower likelihood of physical dysfunction, including upper limb strength (OR = 0.63, 95% CI 0.47, 0.85), lower limb strength (OR = 0.58, 95% CI 0.42, 0.79), and mobility (OR = 0.62, 95% CI 0.45, 0.85) (Table 3). Multiple imputation models showed similar relationships between park availability and physical dysfunction (Appendix Table 1 A). Additionally, a marginal relationship was found between access to utilitarian destinations and lower limb strength (OR = 0.75, 95% CI 0.57, 0.99). Sensitivity analyses using 500- (Appendix Table 1B) and 800-m (Appendix Table 1 C) buffers showed very similar findings, additionally revealing relationships between specific destinations and physical functioning, particularly lower limb strength.

**Table 3:** Relationships of the availability of different neighbourhood destinations within 400-m buffer of participants’ residence with indicators of physical functioning.

	Upper limb strength			Lower limb strength			Mobility		
	OR	(95% CI)	p	OR	(95% CI)	p	OR	(95% CI)	p
Utilitarian destination	0.83	(0.64, 1.09)	0.18	**0.71**	**(0.54**,** 0.95)**	**0.019**	0.92	(0.70, 1.22)	0.57
Public transport station	1.01	(0.84, 1.22)	0.34	0.88	(0.73, 1.05)	0.16	0.96	(0.80, 1.17)	0.70
Park	**0.63**	**(0.47**,** 0.85)**	**0.002**	**0.58**	**(0.42**,** 0.79)**	**0.001**	**0.62**	**(0.45**,** 0.85)**	**0.003**
Sports facility	0.89	(0.65, 1.21)	0.45	1.23	(0.91, 1.66)	0.18	1.03	(0.75, 1.41)	0.86
School	0.91	(0.72, 1.16)	0.45	**0.78**	**(0.61**,** 0.99)**	**0.038**	0.89	(0.69, 1.16)	0.39

OR: odds ratio; CI: confidence interval. Note: Models were adjusted for gender, age group, education, presence of chronic comorbidity, cognitive function, depression, and month for recruitment. Each destination availability was included separately in the models. 95% CIs not include one were highlighted in bold.

Table 4 shows the relationship between destination availability and walking steps. The availability of public transport stations (B = 0.32, 95% CI 0.06, 0.58) and parks (B = 0.47, 95% CI 0.07, 0.87) were associated with more walking steps. The relationships of step counts with physical functioning after adjusting for neighbourhood destination availability were shown in Table 5. More step counts were associated with a lower likelihood of physical dysfunction in upper limb strength, lower limb strength, and mobility. The imputation models showed similar results for the relationship between destination availability and walking steps (Appendix Table 2) as well as the relationship between step counts and physical functioning (Appendix Table 3).

**Table 4:** Relationships of the availability of different neighbourhood destinations within 400-m buffer of participants’ residence with step counts.

	B	(95% CI)	*p*
Utilitarian destination	0.23	(− 0.18, 0.63)	0.27
Public transport station	**0.32**	**(0.06**,** 0.58)**	**0.016**
Park	**0.47**	**(0.07**,** 0.87)**	**0.022**
Sports facility	− 0.29	(− 0.71, 0.13)	0.17
School	0.27	(− 0.07, 0.62)	0.12

B: unstandardised coefficient; CI: confidence interval. Note: Models were adjusted for gender, age group, education, presence of chronic comorbidity, cognitive function, depression, and month for recruitment. Each destination availability was included separately in the models. 95% CIs not include zero were highlighted in bold.

**Table 5:** Relationships of step counts with indicators of physical functioning, after adjusting for exposure (i.e., the availability of specific neighbourhood destination).

	Upper limb strength			Lower limb strength			Mobility		
	OR	(95% CI)	*p*	OR	(95% CI)	*p*	OR	(95% CI)	*p*
Utilitarian destination	**0.79**	**(0.69**,**0.90)**	**< 0.001**	**0.74**	**(0.65**,** 0.85)**	**< 0.001**	**0.74**	**(0.64**,** 0.86)**	**< 0.001**
Public transport station	**0.78**	**(0.68**,** 0.89)**	**< 0.001**	**0.75**	**(0.66**,** 0.86)**	**< 0.001**	**0.74**	**(0.63**,** 0.86)**	**< 0.001**
Park	**0.81**	**(0.71**,** 0.92)**	**0.001**	**0.77**	**(0.68**,** 0.88)**	**< 0.001**	**0.76**	**(0.66**,** 0.88)**	**< 0.001**
Sports facility	**0.78**	**(0.68**,** 0.89)**	**< 0.001**	**0.75**	**(0.66**,** 0.86)**	**< 0.001**	**0.74**	**(0.64**,** 0.86)**	**< 0.001**
School	**0.79**	**(0.69**,** 0.90)**	**< 0.001**	**0.75**	**(0.66**,** 0.87)**	**< 0.001**	**0.74**	**(0.64**,** 0.86)**	**< 0.001**

OR: odds ratio; CI: confidence interval. Note: Models were adjusted for gender, age group, education, presence of chronic comorbidity, cognitive function, depression, month for recruitment, and the corresponding availability of neighbourhood destinations. Each destination availability was included separately in the models. 95% CIs not include one were highlighted in bold.

The joint-significance tests showed some evidence that step counts may partially mediate the relationships of the availability of public transport stations and parks with physical functioning indicators—upper limb strength, lower limb strength, and mobility. However, the mediation effect of walking steps on public transport station availability and physical functioning may be somewhat offset by other factors, as no total effects were found. Sensitivity analyses using imputation models showed similar findings, reinforcing the robustness of the results.

## Discussion

### Main findings of this study

This study examined the relationships between the availability of neighbourhood destinations and indicators of physical functioning in Taiwanese older adults, as well as the potential mediating role of step counts. We found that the availability of parks was associated with a lower likelihood of physical dysfunction, which may be in part mediated by step counts. However, there was no statistical evidence showing an association of the availability of public transport stations with physical functioning. Walking steps appeared to be a potential mediator between the type of destination and physical functioning, but such mediating effects may be offset by other unmeasured factors. Previous studies have shown that the availability or accessibility of destinations is associated with better physical functioning, such as balance^7^ and mobility^8,9^. A greater variety of destinations in residential neighbourhoods may provide venues and increase attractive alternatives for older adults to walk outdoors, engage in physical activity, and interact with neighbours/friends^36^. Our findings extend previous research findings by identifying the relative importance of diverse neighbourhood destinations and highlight park allocation as a key strategy for promoting healthy ageing in communities.

### Comparisons with previous findings

We found that the availability of parks, which are the primary venue for older adults’ daily physical activities^15^, in the residential neighbourhood were specifically associated with increased walking steps and better physical functioning. The weak evidence for walking steps mediating the associations between park availability and physical functioning is consistent with previous findings^13^. Given that older adults tend to be predominantly sedentary^37^, the presence of parks might be more influential for their physical functioning than destinations serving utilitarian or commuting purposes. Leisure-oriented walking, which occurs nearby or within parks, may contribute to longer walking durations than walking for daily errands. A previous study indicated that walking bouts for transport were more likely to be shorter than those for leisure (10–15 minutes vs. 26–40 minutes)^38^.

Differences in the associations between various leisure-oriented locations may be attributable to the equipment provided and accessibility. Parks provide accessible, injury-friendly facilities that cater to diverse users. Therefore, it is easier for older adults to use these facilities without supervision, compared to the professional facilities found in sports facilities such as gyms and stadiums. Additionally, parks, as public open spaces, are designed with features for pacing and areas for rest and aesthetics for the general population. These designs may enhance older adults’ walking experiences in parks. A qualitative study reported that park facilities e.g., benches could enhance the use and enjoyment of green spaces by offering rest points, which can encourage longer walking durations, and positively contribute to older adults’ movement experiences^39^. Another study of walk-along interviews also found walking paths are the most valued park features for walking among the older population^40^. Parks are freely accessible and open around the clock, presenting fewer time or budget limitations for older adults to engage in strolling and walking compared to playgrounds within schools or sports facilities. Further research should investigate older adults’ behavioural patterns in parks to collaborate the mediating role of walking behaviours.

We also found that better availability of public transport stations was associated with more walking steps, which in turn was adversely associated with physical functioning. However, there was no direct association of the availability of public transport stations with physical functioning. This suggests that other behaviours related to public transport use may counteract the benefits of walking. For instance, while public transport users may engage in more walking to and from transit stations^41, they^ also involve longer sitting times during transit^42^. A previous Australian study found that public transport use was linked to both higher daily steps and moderate-to-vigorous physical activity as well as more sedentary time^43^. Additionally, people who rely on public transport often visit a higher number of locations^44^; the positive effects of a higher level of physical activity on health may be trade off against the greater sedentary time accumulated at specific locations such as the workplace and restaurants. Further research should investigate older adults’ behavioural patterns in diverse destinations to better understand other potential mechanisms underlying these relationships.

### Strengths and limitations

This study used objective measures of the availability of neighbourhood destinations, step counts, and indicators of physical functioning. Sensitivity analyses using imputations for missing data and alternative residential neighbourhood destinations were conducted to examine the robustness of the findings. There were some limitations. Participants were recruited from outpatient clinics and community check-ups at a single hospital centre in Taipei, Taiwan. This sample may limit the heterogeneity and generalisability of the results. Future research with a large sample size of older adults from diverse geographic and sociodemographic settings is needed to investigate the associations. Data were collected between September 2020 and March 2021, during the COVID-19 pandemic. The fear of the spread of the virus may have impacted older adults’ willingness to engage in physical activities outside and their daily travel behaviours to these destinations. However, there were no lockdowns or school and work closures, as imposed in many other countries, implemented in Taiwan during the period and thus its impact on physical performance could be assumed negligible. There may be some factors, such as working status and history of exercise activities, that could confound the relationship but were not adjusted for in the models due to data unavailability. However, over 90% of Taiwan’s population aged 65 years or above is retired and non-employed^45^, and more than 85% of them have an exercise habit^15^; thus, the impact on the results could be assumed to be small. Further investigations considering these residual factors can collaborate with the research findings. We examined three measures of physical functioning, including upper and lower limb strength, but not included other functional health indicators such as balance or endurance. Future studies take into account more comprehensive measures of physical functioning are suggested. The study was a cross-sectional design, and therefore, the causal relationships between the availability of neighbourhood destinations and indicators of physical functioning, and the mediation effect of step counts cannot be inferred directly. Future longitudinal studies are needed to examine the causality between these study variables.

### Implications for policy-makers

Our results highlight that the availability of parks was a unique destination providing open spaces for older adults to engage in walking, which in turn contributes to maintaining their physical functioning. These findings offer insights for policy-makers and urban planners in understanding the need to prioritise parks in community designs and create environments that support walking behaviours, ultimately benefiting older adults’ physical functioning.

## Conclusion

Our findings suggest that the availability of parks is associated with a lower likelihood of physical dysfunction in older adults; this association may be partially attributable to more walking step counts. Walking steps may also play a mediating role in the associations of the availability of public transport stations with physical functioning. Spatial allocation and availability of parks that support older adults’ walking behaviours should be considered to maintain or improve their physical functioning. Evidence is needed to explore and clarify the design of parks and the behavioural patterns of older adults in different leisure-oriented destinations that may influence their physical functioning.

## Electronic supplementary material

Below is the link to the electronic supplementary material.

Supplementary Material 1

## Data Availability

Data used in this study are available from the corresponding authors upon reasonable request.
