# Supplementary material for: The mediating role of step counts in the relationship between diverse neighbourhood destinations and older adults’ physical function
Source: Sci Rep. 2025 Apr 11;15:12436. doi: 10.1038/s41598-024-80699-2 (PMC11992067; doi:10.1038/s41598-024-80699-2)
Supplement: Supplementary file 1 — Supplementary Material 1 [file 41598_2024_80699_MOESM1_ESM.docx]

# The Mediating Role of Step Counts in The Relationship Between Diverse Neighbourhood Destinations and Older Adults’ Physical Function

# Appendix Table 1. Relationships of neighbourhood destinations with indicators of physical functioning: sensitivity analyses of (A) using imputation models, (B) defining the neighbourhood as 500 m, and (C) defining the neighbourhood as 800 m

|  | Upper limb strength | | | | |  | Lower limb strength | | | | |  | Mobility | | | | |
| --- | --- | --- | --- | --- | --- | --- | --- | --- | --- | --- | --- | --- | --- | --- | --- | --- | --- |
|  | OR | (95% CI) | | | p |  | OR | (95% CI) | | | p |  | OR | (95% CI) | | | p |
| **(A) using imputation models** | | | | | | | | | | | | | | | | | |
| Utilitarian destination | 0.88 | (0.68 | , | 1.13) | 0.32 |  | **0.75** | **(0.57** | **,** | **0.99)** | **0.039** |  | 0.94 | (0.72 | , | 1.22) | 0.64 |
| Public transport station | 1.04 | (0.89 | , | 1.21) | 0.62 |  | 0.87 | (0.73 | , | 1.02) | 0.085 |  | 0.91 | (0.77 | , | 1.07) | 0.24 |
| Park | **0.71** | **(0.54** | **,** | **0.92)** | **0.011** |  | **0.60** | **(0.45** | **,** | **0.81)** | **0.001** |  | **0.71** | **(0.53** | **,** | **0.95)** | **0.019** |
| Sports facility | 0.92 | (0.70 | , | 1.21) | 0.55 |  | 1.14 | (0.85 | , | 1.52) | 0.39 |  | 1.01 | (0.75 | , | 1.36) | 0.93 |
| School | 0.93 | (0.76 | , | 1.13) | 0.45 |  | 0.84 | (0.68 | , | 1.03) | 0.094 |  | 0.95 | (0.77 | , | 1.18) | 0.66 |
| **(B) defining the neighbourhood as 500 m** | | | | | | | | | | | | | | | | | |
| Utilitarian destination | 0.76 | (0.58 | , | 1.01) | 0.055 |  | **0.68** | **(0.50** | **,** | **0.93)** | **0.014** |  | 0.89 | (0.67 | , | 1.18) | 0.41 |
| Public transport station | 0.91 | (0.76 | , | 1.10) | 0.33 |  | **0.78** | **(0.64** | **,** | **0.94)** | **0.008** |  | 0.88 | (0.73 | , | 1.07) | 0.20 |
| Park | **0.59** | **(0.41** | **,** | **0.86)** | **0.005** |  | **0.59** | **(0.40** | **,** | **0.87)** | **0.008** |  | **0.68** | **(0.47** | **,** | **0.99)** | **0.042** |
| Sports facility | 0.96 | (0.73 | , | 1.26) | 0.78 |  | 1.05 | (0.80 | , | 1.38) | 0.73 |  | 1.03 | (0.78 | , | 1.36) | 0.85 |
| School | 0.93 | (0.70 | , | 1.23) | 0.60 |  | 0.85 | (0.64 | , | 1.12) | 0.24 |  | 1.03 | (0.76 | , | 1.41) | 0.84 |
| **(C) defining the neighbourhood as 800 m** | | | | | | | | | | | | | | | | | |
| Utilitarian destination | **0.67** | **(0.49** | **,** | **0.91)** | **0.009** |  | **0.68** | **(0.49** | **,** | **0.93)** | **0.017** |  | 0.91 | (0.68 | , | 1.23) | 0.91 |
| Public transport station | 0.87 | (0.71 | , | 1.06) | 0.16 |  | **0.81** | **(0.66** | **,** | **0.99)** | **0.042** |  | 0.86 | (0.70 | , | 1.06) | 0.15 |
| Park | **0.37** | **(0.22** | **,** | **0.63)** | **<0.001** |  | **0.44** | **(0.26** | **,** | **0.74)** | **0.002** |  | **0.65** | **(0.44** | **,** | **0.97)** | **0.033** |
| Sports facility | 1.02 | (0.80 | , | 1.29) | 0.88 |  | 1.24 | (0.96 | , | 1.58) | 0.095 |  | 1.15 | (0.90 | , | 1.48) | 0.27 |
| School | 0.78 | (0.53 | , | 1.14) | 0.20 |  | 0.74 | (0.50 | , | 1.08) | 0.12 |  | 0.94 | (0.62 | , | 1.42) | 0.77 |

OR: odds ratio; CI: confidence interval. Note: Models were adjusted for gender, age group, education, presence of chronic comorbidity, cognitive function, depression, and month for recruitment. Each destination availability was included separately in the models. 95% CIs that do not include one were highlighted in bold.

# Appendix Table 2. Relationships of the availability of different neighbourhood destinations within 400-m buffer of participants’ residence with step counts: sensitivity analyses of using imputation models

|  | B | (95% CI) | | | p |
| --- | --- | --- | --- | --- | --- |
| Utilitarian destination | 0.23 | (-0.15 | , | 0.62) | 0.24 |
| Public transport station | **0.26** | **(0.02** | **,** | **0.50)** | **0.036** |
| Park | **0.51** | **(0.13** | **,** | **0.90)** | **0.009** |
| Sports facility | -0.28 | (-0.68 | , | 0.12) | 0.17 |
| School | 0.21 | (-0.12 | , | 0.53) | 0.22 |

B: unstandardised coefficient; CI: confidence interval. Note: Models were adjusted for gender, age group, education, presence of chronic comorbidity, cognitive function, depression, and month for recruitment. Each destination availability was included separately in the models. 95% CIs not include zero were highlighted in bold.

# Appendix Table 3. Relationships of step counts with indicators of physical functioning, after adjusting for exposure (i.e., the availability of specific neighbourhood destination): sensitivity analyses of using imputation models

|  | Upper limb strength | | | | |  | | Lower limb strength | | | | | | | | |  | | Mobility | | | | | | | |
| --- | --- | --- | --- | --- | --- | --- | --- | --- | --- | --- | --- | --- | --- | --- | --- | --- | --- | --- | --- | --- | --- | --- | --- | --- | --- | --- |
|  | OR | (95% CI) | | | p | |  | | OR | | (95% CI) | | | | p | |  | | OR | | (95% CI) | | | | p |  |
| Utilitarian destination | **0.82** | **(0.73** | **,** | **0.93)** | **0.002** |  | | **0.79** | | **(0.70** | | **,** | **0.89)** | **<0.001** | |  | | **0.78** | | **(0.68** | | **,** | **0.89)** | **<0.001** | | |
| Public transport station | **0.82** | **(0.72** | **,** | **0.92)** | **0.001** |  | | **0.80** | | **(0.70** | | **,** | **0.90)** | **<0.001** | |  | | **0.78** | | **(0.68** | | **,** | **0.88)** | **<0.001** | | |
| Park | **0.84** | **(0.75** | **,** | **0.95)** | **0.006** |  | | **0.82** | | **(0.72** | | **,** | **0.92)** | **0.001** | |  | | **0.80** | | **(0.70** | | **,** | **0.91)** | **0.001** | | |
| Sports facility | **0.82** | **(0.72** | **,** | **0.92)** | **0.001** |  | | **0.79** | | **(0.70** | | **,** | **0.90)** | **<0.001** | |  | | **0.78** | | **(0.68** | | **,** | **0.88)** | **<0.001** | | |
| School | **0.82** | **(0.72** | **,** | **0.93)** | **0.002** |  | | **0.80** | | **(0.71** | | **,** | **0.90)** | **<0.001** | |  | | **0.78** | | **(0.68** | | **,** | **0.88)** | **<0.001** | | |

OR: odds ratio; CI: confidence interval. Note: Models were adjusted for gender, age group, education, presence of chronic comorbidity, cognitive function, depression, month for recruitment, and the corresponding availability of neighbourhood destinations. Each destination availability was included separately in the models. 95% CIs not include one were highlighted in bold.
